# Assessing the Reliability of the SICOT Orthopaedic Research Award Selection Process: An Analysis of the Score and the Scorers

**DOI:** 10.7759/cureus.107502

**Published:** 2026-04-21

**Authors:** Shamim Beigh, Jason P Cheung, Rolando Del Cruz, Jishizhan Chen, Sharif Jonayed, Hesham Elbaseet, Mohamed Kenawey, Sureshan Sivananthan, Abdulla K Almahri, Sattar Alshryda

**Affiliations:** 1 Anaesthesia, Al Jalila Children's Speciality Hospital, Dubai, ARE; 2 Orthopaedics, Queen Mary Hospital, Pok Fu Lam, HKG; 3 Premiere Medical Center, Philippine Society for the Surgery of Trauma, Cabanatuan City, PHL; 4 Mechanical Engineering, Torrington Place, University College London, London, GBR; 5 Orthopaedics and Traumatology, National Institute of Traumatology & Orthopaedic Rehabilitation, Dhaka, BGD; 6 Faculty of Medicine, Assiut University Faculty of Medicine, Assiut, EGY; 7 Trauma and Orthopaedics, Royal Manchester Children Hospital, Manchester, GBR; 8 Orthopaedics, ALTY (Adding Life to Years) Orthopaedic Hospital, Kuala Lumpur, MYS; 9 Orthopaedics, Al Jalila Children's Speciality Hospital, Dubai, ARE; 10 Pediatric Orthopaedics and Trauma, Al Jalila Children's Speciality Hospital, Dubai, ARE

**Keywords:** assessor variability, cronbach’s alpha, discrimination index, internal consistency, inter-rater reliability, intraclass correlation coefficient (icc), intra-rater reliability, research award evaluation, scoring criteria, sicot

## Abstract

This study examined the reliability and consistency of the evaluation process used in selecting recipients for the annual Société Internationale de Chirurgie Orthopédique et de Traumatologie (SICOT) Research Award. The primary aim was to assess both inter-rater and intra-rater reliability among the assessors and to evaluate the internal consistency and discriminative capacity of the scoring criteria. A total of 45 anonymised research applications were scored by eight members of the SICOT Research Award Selection Committee. Each application was evaluated across 10 criteria using a 5-point Likert scale, with a maximum total score of 50. The study employed intraclass correlation coefficients (ICCs) to assess reliability, Cronbach’s alpha to measure internal consistency, and discrimination indices to evaluate the performance of individual scoring items.

The results revealed significant variability between assessors. Inter-rater reliability was poor, with ICCs of 0.289 and 0.190 across two evaluation rounds, suggesting inconsistent scoring among different assessors. In contrast, intra-rater reliability, measuring the consistency of individual assessors over time, showed moderate to good agreement, with an ICC of 0.705. The scoring system exhibited excellent internal consistency (Cronbach’s alpha = 0.926), which improved to 0.954 when the "Language" criterion was excluded, indicating that this item may reduce the coherence of the scale. While high internal consistency (Cronbach’s alpha > 0.90) indicates reliability, it may also reflect redundancy and a potential halo effect, and should therefore be interpreted as a limitation rather than a strength. All 10 criteria demonstrated acceptable discriminative power, with the "Aesthetic" item scoring the lowest and the total score showing the highest discrimination index. These findings highlight the strengths and limitations of the current evaluation framework. While the scale itself is internally consistent and capable of differentiating between high- and low-quality applications, inconsistencies in inter-rater scoring point to a need for clearer guidelines and more structured assessor training. The study recommends refining the scoring rubric and exploring AI-based tools to support more objective and transparent evaluation in future selection cycles.

## Introduction

Founded in 1929, Société Internationale de Chirurgie Orthopédique et de Traumatologie (SICOT) is a global leader in trauma and orthopaedics, dedicated to advancing science, improving patient care, promoting research, and fostering international collaboration. The society’s establishment of a Research and Innovation Academy represents a pivotal development in ensuring that SICOT transitions from being solely a conduit of orthopaedic knowledge to also becoming a driving force behind the creation of new evidence-based research [1]. The Society has continuously introduced several research awards to recognise and incentivise scientific excellence, fostering innovation in orthopaedics and related fields.

However, the process of evaluating submissions for such awards is not without its challenges. There is inherent inconsistency in the marking of abstracts and research papers, as highlighted in the literature. Studies have reported variability in scoring due to differences in inter-rater and intra-rater reliability, as well as the subjective biases of assessors [2,3]. Despite the significance of this issue, variability in scoring of award papers remains underreported in the literature. No specific studies to date have systematically examined inconsistency in marking award papers or its impact on the fairness of outcomes.

In this context, it is crucial to emphasise the role of scoring by multiple reviewers to mitigate bias and improve reliability. This study utilised a scoring system involving eight reviewers to ensure a comprehensive evaluation. By relying on assessments from a diverse group of reviewers, rather than only two or three, the study aims to address potential bias and increase the robustness of the evaluation process.

The discussion surrounding the reliability of assessors' evaluations and the need for standardised approaches is particularly important given the influence such processes have on fostering innovation and rewarding excellence. This paper seeks to highlight these challenges, leveraging existing research on grading reliability [4,5] while offering insights into how SICOT’s processes can continue to evolve to ensure fairer, more objective outcomes.

This study evaluates the selection criteria and inter-rater and intra-rater reliability of assessors involved in the selection process for annual research prizes within our orthopaedic society. By identifying potential inconsistencies, the study aims to enhance the evaluation framework to ensure fairness, transparency, and objectivity.

## Materials and methods

A cross-sectional methodological study was conducted during the annual research award selection cycle of 2023. All eight assessors who served on the society’s official evaluation committee during that cycle were included. These assessors were senior academics and clinicians with substantial prior experience in the appraisal of competitive research grant and award applications, ensuring informed and consistent judgment across submissions. Apart from familiarisation with the scoring process and the items being assessed, no formal training was undertaken.

A total of 45 research applications were collected and fully anonymised to eliminate any potential assessor bias related to authorship, institutional affiliation, or prior familiarity with applicants. Each application was independently evaluated by all assessors using a structured selection scale comprising eleven domains. This assessment scale is a generic evaluation tool that has been developed, refined, and progressively evolved by the grant committee over several years to support consistent and transparent appraisal of submissions. The domains assessed were as follows: originality of the topic, complexity of the research question, methodological rigour, significance of the results, clinical relevance, quality of language, aesthetic presentation, structural organisation of the document, overall significance of the project, anticipated impact and future direction, and overall score.

Each domain was scored using a standard, publicly available 5-point Likert scale, ranging from 1 (Poor), 2 (Fair), 3 (Average), 4 (Good), to 5 (Excellent), generating a maximum possible total score of 50 for each application. The Likert scale is a non-proprietary, freely available measurement tool [6]. Descriptive statistics, including means, standard deviations, and score ranges, were calculated for individual domains and total scores using IBM SPSS Statistics for Windows, Version 24 (Released 2016; IBM Corp., Armonk, New York) [7], providing an overview of central tendency and score dispersion.

Inter-rater and intra-rater reliability were evaluated using the Intraclass Correlation Coefficient (ICC). Inter-rater reliability assessed the level of agreement among different assessors, while intra-rater reliability examined the consistency of scoring by the same assessor across applications. ICC values were interpreted according to established thresholds [8,9]: values below 0.5 indicated poor reliability; values between 0.5 and 0.75 indicated moderate reliability; values between 0.75 and 0.90 indicated good reliability; and values exceeding 0.90 indicated excellent reliability.

The internal consistency of the overall selection scale was assessed using Cronbach’s Alpha [10,11]. Alpha coefficients were interpreted using standard criteria, whereby values below 0.5 were considered poor, 0.6-0.7 acceptable, 0.7-0.8 good, and values exceeding 0.8 excellent, reflecting the degree to which the items consistently measured the same underlying construct.

Item discrimination was further examined using two complementary analytical approaches. First, “Cronbach’s Alpha if Item Deleted” was calculated to evaluate the impact of removing each item on the overall internal consistency of the scale. Items whose removal resulted in an increase in the overall alpha value were considered to demonstrate suboptimal discrimination. Second, the Discrimination Index (D) was computed to assess each item’s capacity to differentiate between high-scoring and low-scoring research submissions. A D value of ≥0.30 was considered to represent good discriminative ability, indicating that the item effectively distinguished between stronger and weaker applications [12].

## Results

Descriptive statistics

Eight members of the Research Award Selection Committee participated in the study, scoring the projects based on the specified selection criteria. The committee members scored the applications twice, with a gap of approximately three months between the two scoring sessions. The average score across all applications was 32.11 (SD = 3.39). The item data followed a normal distribution (Kolmogorov-Smirnov test statistic = 0.072). Scores ranged from a minimum of 26 to a maximum of 40.

Inter-rater reliability

Overall inter-rater reliability across the two assessment rounds demonstrated suboptimal agreement. The Intraclass Correlation Coefficient (ICC) for round 1 was 0.289 (95% CI: 0.176-0.450), and for round 2 was 0.190 (95% CI: 0.114-0.304), both indicating poor inter-rater reliability. In contrast, when total project scores were considered, the overall ICC improved to 0.528, reflecting moderate reliability, suggesting that aggregation of multiple domain scores partially mitigated assessor variability.

Item-level analysis revealed considerable variability in inter-rater agreement across individual domains. Agreement was highest for the Methodology domain (ICC = 0.615, 95% CI: 0.387-0.787), indicating moderate to good reliability, while the lowest agreement was observed for Clinical Relevance (ICC = 0.338, 95% CI: −0.015 to 0.613), reflecting poor reliability and wide confidence intervals consistent with assessor disagreement.

Further examination of assessor-level consistency demonstrated extremely poor agreement when individual assessor scores were considered independently. The ICC for single measures was 0.004 (95% CI: 0.002-0.009; p < 0.001), indicating negligible agreement and confirming substantial variability between assessors.

When assessor ratings were averaged, inter-rater reliability improved but remained suboptimal. The ICC for average measures was 0.282 (95% CI: 0.172-0.444; p < 0.001), corresponding to poor to low-moderate reliability (Table 1). These findings indicate that although averaging partially improves consistency, significant inter-assessor variability persists, underscoring the need for improved calibration, clearer scoring anchors, and further refinement of the evaluation rubric. 

**Table 1:** Intraclass Correlation Coefficient of Assessors This table presents the Intraclass Correlation Coefficients (ICC) for inter-rater reliability of the research award selection scale across two independent assessment rounds and for the overall dataset. Single-measure ICC reflects the reliability of individual assessor ratings, while average-measure ICC reflects the reliability of the mean scores derived from all assessors. ICCs were calculated using a two-way mixed-effects model with absolute agreement definition. Corresponding 95% confidence intervals, F statistics, degrees of freedom (df1 and df2), and significance levels are reported. Values are interpreted according to standard reliability thresholds, where higher ICC values indicate greater inter-rater agreement. ^a^The estimator is the same, whether the interaction effect is present or not. ^b^Type A intraclass correlation coefficients using an absolute agreement definition. ^c^This estimate is computed assuming the interaction effect is absent, because it is not estimable otherwise.

Rounds	Items measured	Intraclass Correlation^b^	95% Confidence Interval		F Test With True Value 0			
			Lower Bound	Upper Bound	Value	df1	df2	Sig
Round 1	Single measures	.005^a^	.002	.009	8.284	27	2349	.000
	Average measures	.289^c^	.176	.455	8.284	27	2349	.000
Round 2	Single measures	.003^a^	.001	.005	6.311	39	3393	.000
	Average measures	.190^c^	.114	.304	6.311	39	3393	.000
Overall	Single measures	.123^a^	.044	.255	2.598	29	203	.000
	Average measures	.528^c^	.268	.733	2.598	29	203	.000

Intra-rater reliability

The overall ICC for intra-rater reliability was 0.705 (95% CI: 0.414-0.995), and it ranged from 0.174 (95% CI: -0.368-0.523) to 0.992 (95% CI: 0.986-0.996), suggesting moderate to good agreement.

Item discrimination

The selection scale demonstrated excellent overall internal consistency, with a Cronbach's Alpha of 0.926 (95% CI: 0.874-0.950). Analysis using Cronbach's Alpha if Item Deleted revealed that the item "Language" was scored inconsistently. Removing this item improved the scale's internal consistency, increasing Cronbach's Alpha to 0.954 (95% CI: 0.923-0.970). Analysis using the Discrimination Index to compare top-scoring and bottom-scoring projects showed that all items demonstrated strong discrimination, with values ≥0.30. The lowest Discrimination Index was 0.68 for the "Aesthetic" item, while the highest value was 8.27 for the overall average total score (Table 2). 

**Table 2:** Item Analysis and Internal Consistency of the Research Award Assessment Scale This table presents the results of the item-level reliability analysis of the research award selection scale. For each item, the scale mean and variance if the item were deleted, the corrected item–total correlation, and Cronbach’s alpha if the item were deleted are reported. Corrected item–total correlation values reflect each item’s contribution to the overall scale consistency, with higher values indicating stronger discrimination. Cronbach’s alpha, if an item is deleted, demonstrates the impact of removing individual items on the overall internal consistency of the scale.

Items	Scale Mean if Item Deleted	Scale Variance if Item Deleted	Corrected Item-Total Correlation	Cronbach's Alpha if Item Deleted
Originality	28.9272	10.048	.674	.921
Complexity	28.9562	9.768	.799	.914
Methodology	28.9380	9.343	.785	.915
Signficant_of_result	28.9698	9.661	.882	.911
Clinical_Revelance	28.8061	9.847	.860	.913
Language	28.6373	9.688	.395	.954
Aesthetic	29.0875	10.213	.711	.919
Structure	28.9350	9.935	.738	.917
Overall_Signficance	28.8706	9.743	.901	.911
Impact_on_Future_Direction	28.8995	9.676	.895	.910

## Discussion

This study aimed to evaluate the inter-rater and intra-rater reliability of assessors in the context of the annual research award selection process conducted by SICOT. By identifying inconsistencies and analysing item discrimination, the study sought to improve the fairness and transparency of the evaluation framework [13].

The scoring of 45 anonymised research applications by eight assessors showed an average score of 32.11 (SD = 3.39), with scores ranging from 26 to 40. The data distribution was normal (Kolmogorov-Smirnov test statistic = 0.072). Given that the total possible score per application is 50, an average of 32 was reasonable.

The overall inter-rater reliability, as measured by ICC, was poor in both scoring rounds (Round 1: 0.289; Round 2: 0.190). The reliability for individual items varied, with "Methodology" demonstrating the highest inter-rater reliability (ICC = 0.615) and "Clinical Relevance" the lowest (ICC = 0.338). Intra-rater reliability showed moderate to good agreement, with an overall ICC of 0.705 [14, 8, 9, 15].

Several factors may account for the poor inter-rater agreement observed in the study. Assessors likely varied in their levels of expertise, and some may have lacked familiarity with the scoring process. Without standardised training or calibration sessions, differences in how assessors interpreted the scoring guidelines could have compromised consistency. Additionally, the complexity of evaluating multiple dimensions (e.g., originality, methodology, aesthetics) might have introduced cognitive biases or inconsistencies in how individual criteria were weighted [3].

While it was initially suspected that the scoring criteria might not be well defined, potentially allowing for subjective interpretation, the selection scale demonstrated excellent internal consistency (Cronbach’s Alpha = 0.926) [16, 10]. However, inconsistent scoring was evident for specific items, such as "Language." Removing this item improved internal consistency (Cronbach’s Alpha = 0.954), highlighting its variability. The Discrimination Index further showed strong discrimination for all items (≥0.30), with "Aesthetic" having the lowest value (0.68) and the overall score showing the highest discrimination (8.27).

Our study possesses several strengths and is grounded in a clear and important objective. It provides a comprehensive evaluation of inter-rater and intra-rater reliability, while also examining the internal consistency and discriminatory capacity of individual items. Importantly, the analysis is based on real-world applications assessed by practising evaluators, rather than hypothetical or simulated data, enhancing its practical relevance. The inclusion of eight assessors and 45 applications represents a relatively robust sample size for this type of study, and the use of established statistical measures, including ICC and Cronbach’s Alpha, supports the methodological rigour of the analysis.

However, we acknowledge that the high internal consistency observed (Cronbach’s Alpha > 0.90), while indicative of reliability, may also suggest redundancy among the scoring domains and the potential presence of a "halo effect," whereby assessors rely on a general impression rather than fully discriminating between individual criteria. This finding should therefore be interpreted with caution and viewed as a limitation of the current framework rather than a definitive strength.

By identifying both the variability in scoring and the potential overlap between domains, this study highlights important areas for refinement. These insights provide a foundation for improving the structure and clarity of the scoring system, ultimately contributing to a more transparent, discriminative, and equitable evaluation process.

The main limitation of our study is that variability in assessor expertise and familiarity with the criteria may have influenced the results. Moreover, while the selection criteria and inter-rater and intra-rater reliability were assessed, external factors affecting consistency, such as scoring guidelines or training, were not explored.

The findings highlight significant variability in inter-rater reliability, emphasising the need for clearer scoring criteria and enhanced assessor training. Poor agreement among assessors could undermine the credibility of the selection process, necessitating standardisation efforts to reduce subjective interpretation. The good intra-rater reliability indicates that individual assessors were generally consistent over time, but variability in certain items suggests the need to refine specific criteria, such as "Language."

The high internal consistency of the scale and strong discrimination across all items validate its overall reliability as an evaluation tool. However, the inconsistent scoring of the "Language" item highlights the importance of reviewing and potentially removing or redefining criteria that contribute to inconsistency.

Integrating AI into the selection process could address challenges such as subjectivity, inconsistency, and inefficiency, leading to a more robust, transparent, and equitable evaluation process [17]. AI can also help standardise the interpretation of scoring criteria by providing clear, data-driven guidelines for each evaluation dimension. This minimises subjectivity and ensures that assessors apply consistent standards. This will be the subject of our next research project on research award selection.

## Conclusions

This study provides valuable insight into the reliability and fairness of the SICOT research award selection process. Although the assessment scale demonstrated strong internal consistency and good item discrimination, inter-rater reliability was poor, indicating notable variability in how assessors interpreted and applied the evaluation criteria. In contrast, intra-rater reliability was moderate to good, suggesting that individual assessors were generally consistent in their scoring over time.

The inconsistent performance of certain items, particularly “Language,” indicates the need for refinement or potential removal of criteria that introduce scoring variability. Future enhancements should include clearer scoring definitions, structured assessor calibration sessions, and consideration of AI-assisted support tools to reduce subjectivity. Such measures would strengthen the objectivity, fairness, and credibility of the SICOT award selection process.
